# A Simulation Optimization Approach for Resource Allocation in an Emergency Department Healthcare Unit

**DOI:** 10.5334/gh.528

**Published:** 2020-02-11

**Authors:** Arman Bahari, Farzaneh Asadi

**Affiliations:** 1 Faculty of Industry and Mining, University of Sistan and Baluchestan, Zahedan, IR; 2 Department of Engineering, Islamic Azad University, Branch of lahijan, IR

**Keywords:** Emergency, Simulation Models, Resource allocation, Factor Design

## Abstract

**Background:**

Effective Decision Making on the resources of the ED plays a significant role in the performance of the department. Since wrong decisions can have irreparable consequences on the quality of services, the decision-makers should analyze and allocate the resources effectively.

**Methods:**

The present study aimed to investigate the effective resources in the emergency department and provide an optimal combination of these resources based on the meta-modeling optimization approach to reduce the wait time for patients in the ED.

**Results:**

The results demonstrated that the number of CHWs and beds played a significant role in the total average wait time for patients. Although the effect of other variables was not statistically significant, they were deliberately used in this study to determine the optimal combination of such variables by solving the problem.

**Conclusion:**

The findings of the present simulation-model approach provide hospital managers with valuable data in order to control and re-design the admission to discharge procedure in the emergency in order to enhance efficiency. By considering the budget, the new configuration of 2 Community Health Worker, 1 Receptionist, 1 nurses, 3 Cardiologist and 10 beds, with 142 minutes of a patient’s wait time shows 49.6% wait time improvement and a reduction of 51% in the cost of resource usage.

Recently, the pressure on the emergency department (ED) as the main entrance of hospitals has been raised, due to the increasing demands related to different factors (such as population growth), which has led to patient dissatisfaction [1,2,3,4]. Due to patient dissatisfaction with hospital services, high costs and growing demands for healthcare, field managers attempt to decrease costs and increase efficiency by allocating optimal resources. Further, the state laws of many countries, especially Iran, require healthcare systems to provide their services at high qualities in the minimum time. To this end, field researchers have focused on applying a combination of service providers and scarce resources in different sections of health care centers to determine the criteria of desired performance in certain conditions and restrictions [5].

Making a decision on ED resources plays an important role in the performance of the department. Since wrong decisions can have irreparable consequences on the quality of services, decision-makers should analyze the hospital system and allocate resources effectively. This kind of analysis is difficult due to the stochastic environment of the ED, and has a time-dependent behavior, leading to major variations in arrival rates of patients and service rates in most cases over time [6].

The present study aimed to investigate the effective resources in the emergency department, and to provide an optimal combination of these resources to reduce the wait time for patients in the ED, based on the meta-modeling optimization approach. The model was implemented through response surface methodology and the design of experiments. In the current study, the total average wait time for patients in the ED was considered as the dependent variable in which the response surface was made through conducting 32 experiments created by factorial design in the simulation model and implemented for five key variables in both maximum and minimum levels. Community health workers (X_1_), receptionists (X_2_), nurses (X_3_), cardiologists (X_4_), and ED beds (X_5_) were considered as independent variables. The inputs required to create the model included the amount of allocating different patients to each part of the process, the amount of the costs allocated to the resources and the number of required patients to repeat the simulation.

Since resource planning is crucial for reducing the average wait time for patients in the intensive care unit, the present study aimed to simulate the process-based approach, while eliminating the public sector in the model. In the simulation model, the process begins when a patient arrives through the ED, and ends when a patient is discharged from the ED or is admitted into hospital inpatient units. The triage system in the ED is based on the urgency in which the patients are categorized from 1 (most urgent) to 5 (least urgent) levels. This department receives within 24 hours an average of 52,000 patients a year, which is about 145 patients daily. About 40% of patients of this ED have heart problems and should receive their services as soon as possible [4].

The Figure 1 below illustrates the simulation flow of patients in the form of a process simulation in Visual Paradigm International Ltd 12.0. As seen in the figure, based on the classification, ESI 1 refers to patients with the highest urgency, usually admitted to the hospital by ambulance. These patients are promptly transported to the CPR center where they are resuscitated via ECG. In the case of successful CPR, the patient is transported to the CPU. ESI 2 patients are primarily physically assessed by nurses. Afterwards, they pass the admission section and enter the CPU with no delay. ESI 3–5 patients should first go to the admission section, where their personal information is recorded. After the admission procedure, ESI 3 and 4 patients are transported to the intensive care unit, while ESI 5 patients leave the emergency after visiting general practitioners.

**Figure 1:**
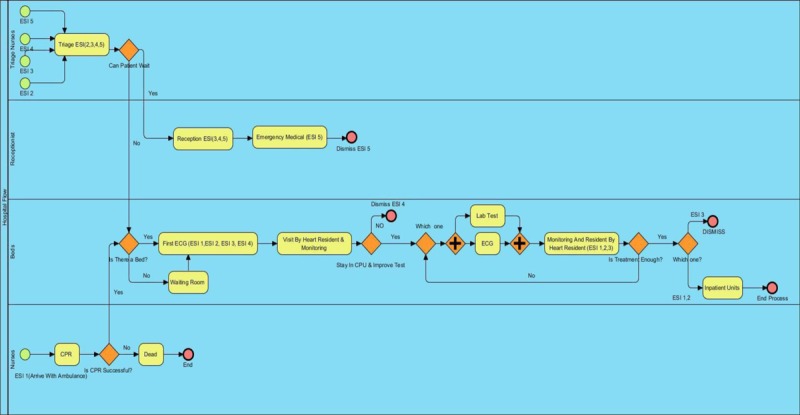
The simulated flow of patients in the ED, by Visual Paradigm.

According to the obtained results from statistical tests of validation, which are collected for a regression meta-model with different interactions, a response-surface meta-model with three different interactions (P-Value = 0/0483<0/05) was selected as the candidate meta-model. It means only trinary combinations of the main variables were used in the meta-model, including: *X*_1_*X*_2_*X*_3_, *X*_1_*X*_2_*X*_4_, *X*_1_*X*_3_*X*_5_, *X*_1_*X*_4_*X*_5_, *X*_2_*X*_3_*X*_4_, *X*_2_*X*_4_*X*_5_, *X*_3_*X*_4_*X*_5_. The quadratic and quintuple combinations were excluded. Table 1 indicates the statistical analysis and fitting of the experiment factors. As shown, the factors with p-value < 0.05 remained in the meta-model. In the experiments related to the wait time of patients, the amount of R^2^ was 94.19%, which indicated the accuracy of variations in the rate of patient wait time. The adjusted-R^2^ showed 70% variation in the independent variables of the model, which are the number of key resources. The sum of squares of the predicted errors for the model was 2.94. Also, obtaining the statistics for controlling the efficiency of the predictions due to the proper performance of the model is in the prediction phase.

**Table 1:** Estimation of interactions and coefficients for patient wait time.

Resource	Sum of Squares	Interaction Estimation	Standard Error	F-Value	P-value

**Model**	1.67	0.21	0.023	3.89	0.0483
***X*_1_**	0.21	–0.08	0.023	12.05	0.0133
***X*_2_**	0.039	0.035	0.023	2.28	0.1815
***X*_3_**	0.040	0.035	0.023	2.30	0.1802
***X*_4_**	0.035	–0.033	0.023	2.04	0.2034
***X*_5_**	0.17	–0.073	0.023	9.91	0.0199
***X*_1_*X*_2_**	0.004	–0.012	0.023	0.26	0.6277
***X*_1_*X*_3_**	0.091	–0.053	0.023	5.29	0.0610
***X*_1_*X*_4_**	0.084	–0.051	0.023	4.89	0.0690
***X*_1_*X*_5_**	0.16	0.071	0.023	9.39	0.0221
***X*_2_*X*_3_**	0.072	–0.047	0.023	4.16	0.0857
***X*_2_*X*_4_**	0.041	–0.036	0.023	2.38	0.1737
***X*_2_*X*_5_**	0.013	–0.02	0.023	0.73	0.4249
***X*_3_*X*_4_**	0.005	–0.013	0.023	0.30	0.6031
***X*_3_*X*_5_**	0.21	0.081	0.023	12.27	0.0128
***X*_4_*X*_5_**	0.006	–0.004	0.023	0.038	0.8515
***X*_1_*X*_2_*X*_3_**	0.086	–0.052	0.023	5.02	0.0662
***X*_1_*X*_2_*X*_4_**	0.004	–0.012	0.023	0.28	0.6136
***X*_1_*X*_2_*X*_5_**	0.11	0.057	0.023	6.13	0.0481
***X*_1_*X*_3_*X*_4_**	0.049	–0.039	0.023	2.89	0.1417
***X*_1_*X*_3_*X*_5_**	0.16	0.071	0.023	9.29	0.0226
***X*_1_*X*_4_*X*_5_**	0.036	–0.034	0.023	2.12	0.1961
***X*_2_*X*_3_*X*_4_**	0.010	–0.018	0.023	0.59	0.4707
***X*_2_*X*_3_*X*_5_**	0.041	–0.036	0.023	2.36	0.1756
***X*_2_*X*_4_*X*_5_**	0.003	–0.01	0.023	0.2	0.6711
***X*_3_*X*_4_*X*_5_**	0.003	–0.009	0.023	0.18	0.6841

*R*^2^ = 0/9419, *adjusted – R*^2^ = 0/70, PRESS = 2/94, Adeq – Precisior = 9/020.

The results of p-value analysis are as follows. The main factors *X*_1_ and *X*_5_ are statistically significant, while *X*_2_, *X*_3_ and *X*_4_ are not. The interaction effects between *X*_1_ and *X*_5_; *X*_5_ and *X*_3_ are significant, statistically. However, other two-way interaction effects between variables are not as such. In addition, the internal effects of X_2^2 are significant statistically, while those of X_1^2 and X_3^2 are not significant.

Based on the analysis, statistically significant factors were included in the pseudo-model, while those of statistic insignificance were deleted in the final model. The decision variables of *X*_2_, *X*_3_ and *X*_4_ are intentionally applied to the pseudo-model, despite statistic insignificance, considering minor errors. Therefore, the nonlinear objective function can be fitted using the regression model with three interactions according to equation 1.

1 \begin{array}{l} Y = 0/21 - 0/08{X_1} + 0/035{X_2} + 0/035{X_3} - 0/033{X_4} - 0/073{X_5}\\ \;\;\;\;\;\,\,\;\;\;\; + \;0/071{X_1}{X_5} - 0/081{X_3}{X_5} + 0/057{X_1}{X_2}{X_5} + 0/071{X_1}{X_3}{X_5} \end{array}

The meta-model presented in equation 1 was implemented in the ED of the hospital to find the optimal number of key resources for reducing patient wait time within management constraints. The general mathematical structure for optimizing the resources was based on equation 2.

2 min\;Y = f\left({{X_1},\;{X_2},\;{X_3},\;\;{X_4},\;{X_5}} \right)

Subject to

**Table:** 

*L_i_* ≤ *X_i_* ≤ U*_i_*	*For*	*i* = 1, 2, 3, 4, 5
\sum\nolimits_{i = 1}^5 {{C_i}{X_i} \le B}	*For*	*i* = 1, 2, 3, 4, 5
*X_i_ Integer*	*For*	*i* = 1, 2, 3, 4, 5

*Y = The optimal combination of the ED resources with X*_1_, *X*_2_, *X*_3_, *X*_4_, *X*_5_ *variables*.

*L_i_ and U_i_ = The minimum and maximum acceptable level of resource capacities*.

*X_i_ = The decision variable or the effective resources in the ED*.

*C_i_ = The cost of using each resource*.

*B = The available budget for the resources determined by the management and obtained by the sum of the costs of each resource*.

The model represents an optimization problem in which Y is the optimal combination of emergency resources to reduce patient wait time, and is a function of X_1_, X_2_, X_3_, X_4_, and X_5_ variables. This function acts as a black box, which is estimated by simulation, design and analysis of experiments with the desired accuracy. Establishing the optimal combination created by solving the model can minimize the wait time for patients. The parameters specified for all variables in this study, are shown in Table 2.

**Table 2:** Parameters specified for key variables.

Decision variables	L_*i*_	U_*i*_	Cost

(***X*_1_**)	1	2	0.6
(***X*_2_**)	1	3	0.5
(***X*_3_**)	1	6	0.8
(***X*_4_**)	1	3	0.7
(***X*_5_**)	5	10	2

Table 3 shows a comparison between current and proposed status, which was simulated for 381 patients over a working day.

**Table 3:** Comparing the current and proposed status.

Status	Decision Variable					Wait Time (minutes)	Costs ($)

	Community Health Worker	Receptionist	Nurse	Cardiologist	Bed		

**Current**	1	2	3	1	7	282	4140000
**Proposed**	2	1	1	3	10	142	1980000

The results demonstrated that the number of CHWs and beds played a significant role in the total average wait time for patients. Although the effect of other variables was not statistically significant, they were deliberately used in this study to determine the optimal combination of such variables by solving the problem. The results indicated a 49.6% decrease in total wait time of patients through replacing the combination of the proposed resource. Making such a decision is vital for management since the wait time of patients in ED, especially the most urgent ones, should be reduced as much as possible. In the proposed model, the optimal number of community health workers, receptionists, nurses, cardiologists, and rooms were determined as two, one, one, three, and ten, respectively. These changes reduced the costs of the system from $4,140,000 to $1,980,000. Accordingly, when resources were allocated in ED based on the proposed model, this model provided a 49.6% improvement in the wait time of patients (reduced from 4 hours and 42 minutes [282 min] to 2 hours and 22 minutes [142 min] in a working day).

The reduction in wait time (from 282 to 142 min) in the model of this research is satisfactory compared to the study conducted by Zeinali et al. [4] who managed to implement a 48% reduction in the total wait times of patients (reducing the time from 44 to 23 min), subject to both budget and capacity, in an emergency department in Iran. Moreover, this model can provide a 51% improvement in the level of existing costs, while the study of Zeinali et al. [4] managed to provide a 49% improvement. Also, compared to another study performed by Dengiz et al. [7] a better regression meta-model was offered due to their sufficient consideration of statistical analyses. They developed a production control model for a paint shop department in an automotive company in Turkey. Dengiz et al. [7] employed a regression meta-model with three interaction effects in their study and only provided a 15% improvement (maximum number of buses painted daily (10.475 buses)) in the investigation process. Thus, in the present research, a better level of improvement (51%) in the main study objective was obtained compared to this regression meta-model.

The findings of the present simulation-model approach provide hospital managers with valuable data to control and re-design the admission-to-discharge procedure in the emergency department, in order to enhance efficiency. By considering the budget, the new configuration of 2 community health workers, 1 receptionist, 1 nurse, 3 cardiologists and 10 beds, with 142 minutes of patient wait time, shows a 49.6% wait time improvement and a reduction of 51% in resource usage.

Based on the findings of this study, several recommendations are provided to improve the performance of this emergency department. a) Increase accuracy and improve timing of patient services to reduce losses and resources. To do this, community health workers, receptionists, nurses and cardiologists need to be on time. Also the required information (patient records and test results) has to be available at the right time. b) Re-evaluate emergency department scheduling to ensure that the process time is accurate. We recommend health care managers collect and record process data on emergency department processes systematically to develop decision support systems, as well as to monitor and evaluate the processes and resources of the ED. Of course, we know that data collection is not easy, and will require working groups and the use of information technology. c) Managers of the ED can evaluate the wait time of patients within the constraints of budget to decide whether there should be an increase in budget for a special performance level, or that the patient should spend more time in the system and accept this service because of budget limitations.

We believe that the proposed model helps managers save time, money, and resources, and is the foundation to reduce patient wait time. Also the model of this study can be easily reused, with minor changes, in other emergency departments in Iran.

Further research can be conducted for determining the optimal combination of resources to solve other complicated issues in real-life by applying the presented method, and by using other meta-models such as Kriging and neural network. Also, it is recommended that the results of the suggested studies by considering one problem with more than one objective should be compared with the results of the present study.
